# Bacterial Nanocellulose—A Biobased Polymer for Active and Intelligent Food Packaging Applications: Recent Advances and Developments

**DOI:** 10.3390/polym12102209

**Published:** 2020-09-26

**Authors:** Karolina Ludwicka, Monika Kaczmarek, Aneta Białkowska

**Affiliations:** Institute of Molecular and Industrial Biotechnology, Lodz University of Technology, B. Stefanowskiego 4/10, 90-924 Lodz, Poland; monika.kaczmarek@dokt.p.lodz.pl (M.K.); aneta.bialkowska@p.lodz.pl (A.B.)

**Keywords:** bacterial nanocellulose, food packaging, active packaging, intelligent packaging, biopolymer, composite, biosensor

## Abstract

The aim of this review is to provide an overview of recent findings related to bacterial cellulose application in bio-packaging industry. This constantly growing sector fulfils a major role by the maintenance of product safety and quality, protection against environmental impacts that affect the shelf life. Conventional petroleum-based plastic packaging are still rarely recyclable and have a number of harmful environmental effects. Herein, we discuss the most recent studies on potential good alternative to plastic packaging—bacterial nanocellulose (BNC), known as an ecological, safe, biodegradable, and chemically pure biopolymer. The limitations of this bio-based packaging material, including relatively poor mechanical properties or lack of antimicrobial and antioxidant activity, can be successfully overcome by its modification with a wide variety of bioactive and reinforcing compounds. BNC active and intelligent food packaging offer a new and innovative approach to extend the shelf life and maintain, improve, or monitor product quality and safety. Incorporation of different agents BNC matrices allows to obtain e.g., antioxidant-releasing films, moisture absorbers, antimicrobial membranes or pH, freshness and damage indicators, humidity, and other biosensors. However, further development and implementation of this kind of bio-packaging will highly depend on the final performance and cost-effectiveness for the industry and consumers.

## 1. Introduction

The packaging industry is currently a thriving market that plays an important role in a modern economy. Several factors, such as globalization, innovative technologies, and increasing consumer requirements highly affect and stimulate the development of this sector. Among others, food and beverages occupy the largest segment of this market (85%) [1]. Nowadays, petroleum-based products (e.g., polyethylene, polypropylene) are widely used in food packaging industry, although they are well known to have negative impact on ecosystem. Therefore, there is a huge potential laying in bio-based relatives, as they are biodegradable, do not adversely affect the climate, and eliminate the risk of cross-contaminations during recycling, as well as danger of toxicity to consumers [2]. The increasing concerns for the environmental pollution and ecological imbalances caused by expansive usage of ecologically nonfriendly materials have led to the growing awareness and the development of new natural, green alternatives to plastic packaging. In recent years, the growing number of eco-products on the market has opened a new perspective for bio-based products despite their higher price [3]. Numerous biopolymers may be found both in the literature as well as on market shelves. At the same time, the new trends of innovative active and intelligent packagings development direct the research on biopolymers adjustment and modification to produce these eco-products with highly advanced functions and properties. Among them, one of the most attractive is bacterially synthesized nanocellulose (composites or native material), which has been used as a natural bio-substrate for such applications as medicine and cosmetics but recently also in packaging industry and electronics. In this manuscript, an overview of the most recent findings on bacterial cellulose usage specifically as an active and/or intelligent food packaging are provided. The limitations and future perspectives related to this issue, highlighting the advantages of this microbial polysaccharide over other biopolymers, are also briefly mentioned.

Bacterial nanocellulose (bionanocellulose, BNC, bacterial cellulose, BC, microbial cellulose, MC) is a linear, unbranched exopolysaccharide synthesized by some bacteria, which consist only of β-D-glucopyranose units linked by β-1,4-glycosidic bonds. The structure of this polymer consists of ultrafine nanofibrils, which form a three-dimensional web-shaped construction stabilized by inter- and intramolecular hydrogen bonds. The molecular formula of bacterial nanocellulose (C_6_H_10_O_5_)_n_ is the same as that of plant origin, but its physicochemical properties are different. Compared to plant cellulose, BNC is characterized by considerably higher crystallinity (84–89%), excellent chemical purity (lack of impurities such as hemicellulose, lignin, or pectin), high tensile strength in comparison to other biomaterials, and good moldability [4]. Additionally, bacterial nanocellulose is characterized by a smaller cross-section of fibers, which contributes to high porosity of this material [5,6]. Moreover, BNC production does not require harsh chemical treatments for cellulose isolation and purification [7]. This biopolymer is synthesized by many bacteria, including *Komagataeibacter* (former *Gluconacetobacter*), *Agrobacterium, Achromobacter, Alcaligenes, Aerobacter, Pseudomonas, Rhizobium, Dickeya, Rhodobacter,* and *Sarcina* [8]. However, the main and the most efficient, as well as widely investigated, producers of BNC include Gram-negative, aerobic bacteria belonging to the *Komagataeibacter* genus [9,10]. These acetic acid bacteria are capable of metabolizing various carbon sources, such as glucose, fructose, mannitol, xylose, glycerol, dihydroxyacetone, or dicarboxylic acids into linear β-(1–4)-glucan chains and then, finally, extrude them through multiple pores located in their cytoplasmic membrane [4,11]. The chemical properties of bionanocellulose are, therefore, primarily associated with the construction of a repeating unit—cellobiose, which contains three free hydroxyl groups in positions C2 and C3 (secondary alcohols) and C6 (primary alcohol). Due to their presence, BNC possesses a chemically reactive surface and relatively easily undergoes etherification, esterification, and acylation reactions. Additionally, microbial cellulose is a non-toxic and naturally porous biomaterial, which exhibits unique properties including remarkable biocompatibility, biodegradability, strong hydration, and excellent water-holding capacity (over 100 times of its own weight) [6,11]. The fibrillated network of the BNC results in very good mechanical properties, such as high elasticity (Young modulus of 15–18 GPa) and tensile strength, which places this material in a very high position over other natural polymers [4]. All the properties of bacterial cellulose strongly depend on many factors, including pH and composition of the culture medium, cultivation time and conditions, as well as selection of the producing microorganism [12]. Nevertheless, the membrane itself finds numerous fields of its applications, mainly medicine and cosmetic ones, but also the food industry where it has been known for years as a dessert from Philippines called *Nata de coco* [12]. In the latter sector, it has been recently applied and specifically modified to constitute a natural, bacterially produced packaging [13].

## 2. The Market of Bio-Based Packagings

Packaging is an integral component of food industry and plays an important role in providing protection from chemical, biological, and physical challenges by acting as a boundary between food and the surrounding environment. This type of material has several functions, from preventing product breakage to providing a barrier against moisture, oxygen, carbon dioxide, radiation/light, as well as aromas and flavors. In addition, packages are designed to ensure microbial safety and a proper shelf life of food. The packaging industry currently uses various petroleum-derived plastics, glass, metal, paper, aluminum, and increasingly, biopolymers as raw materials [14]. Synthetic polymers have had a major share in this sector due to their excellent mechanical properties, transparency, flexibility, thermoplasticity, ease, and low cost of production. However, despite these advantages, a growing number of petroleum-derived materials constitute a serious environmental problem. Conventional food packagings are resistant to degradation, which causes the accumulation of plastic waste and leads to pollution of the ecosystem. Other disadvantages of synthetic polymers include high CO_2_ emissions during production, risk of carcinogenic diseases (some additives present in plastics are potentially toxic), and high recycling costs [2]. According to the estimates provided by the European Commission, approximately 25.8 million tons of plastic waste are generated annually in Europe and only less than 30% of them are recycled [15]. Therefore, European Green Deal launched a concerted strategy leading to achieve climate neutrality by 2050. The EU Strategy for Plastics in a Circular Economy assumes, inter alia, developing the concept of packaging for re-use and recycling, including the implementation of green alternatives or reusable systems. The European Commission decisions regarding the issue of environmental waste direct to an increased interest in the development of biodegradable packaging materials such as bacterial nanocellulose [16].

**Figure 1:**
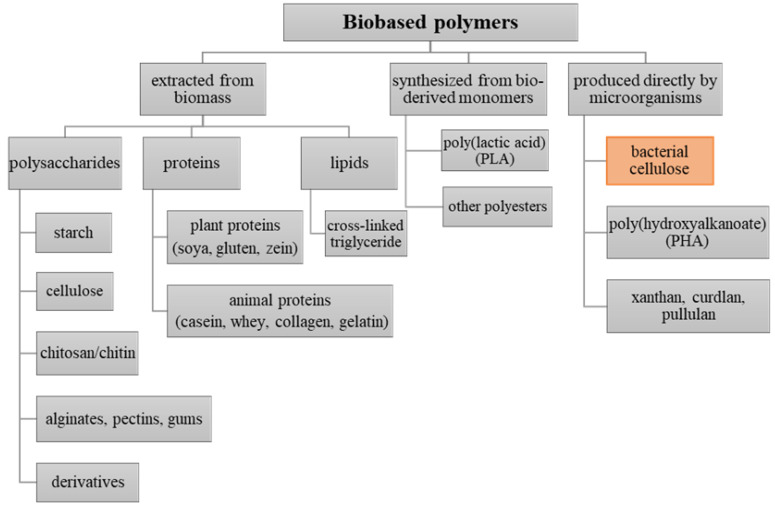
Schematic overview of bio-based packaging materials categorized by their origin and method of production [54,55].

The so-called *biopackaging* is made of material derived from renewable sources, which fully decompose. It can be directly synthesized by biological systems (e.g., plants, animals, algae, microorganisms) or through the polymerization of biobased monomers (e.g., polylactic acid). Accordingly, these polymers may be divided into three main categories depending on their origin and method of production (see Figure 1) [17]. Such materials, based on renewable resources that are usually utilized for food packaging applications include, i.a., polysaccharides (e.g., cellulose, starch, chitosan) and proteins (e.g., collagen, casein, gluten) extracted from biomass, polymers synthesized from bio-derived monomers (e.g., polylactic acid (PLA)), as well as those produced directly by microorganisms (e.g., polyhydroxyalkanoates (PHA), bacterial cellulose, pullulan, curdlan, xanthan) [18].

Natural polysaccharides, proteins, and their derivatives are one of the biopolymers used in the packaging industry [19]. These bio-based materials have relatively good barrier properties and can be manufactured on a large industrial scale at moderately low costs, which makes them an attractive substitute for petroleum-derived plastics. However, commercialization of polymers extracted from biomass is still limited due to high divergence in relation to plastics, most of all poor tensile strength, brittleness, thermal instability, and water sensitivity [20,21,22,23]. Therefore, in order to obtain edible films and coatings of improved quality, they are blended with various reinforcing materials and compounds such as plasticizers (e.g., glycerol, glycol, sorbitol) [24,25,26,27,28,29].

Among naturally derived biopolymers, those extracted from a biomass constitute one of the major categories of biobased, industrially relevant molecules. In this group, alginates and chitosan have been recognized as highly valuable due to their natural origin and very useful properties. Alginate is a polysaccharide isolated from brown algae (its monomers are α-L-guluronic acid and (1,4)-linked β-D-mannuronic acid) and have been widely used for producing films, gels, suspensions, and emulsions for food applications [30]. It known for its relatively low price (as for a natural polymer), as well as for the ability of functional groups to react with polyvalent cations. Most often, alginates are crosslinked for improvement of their resistance in water, mechanical properties, and coherence [31]. Chitosan, extensively investigated especially in the medical sector, is a known natural bio-active polymer with an immanent antimicrobial activity, which promotes its usage as a film or coating, for preservation and storage of numerous foods, mainly fruits and vegetables [32]. This polysaccharide composed of randomly distributed β-(1–4)-linked D-glucosamine (deacetylated unit) and N-acetyl-D-glucosamine (acetylated unit) is derived mainly from chitin shells of crustaceans, but is also synthesized by some fungi belonging e.g., to *Mucoraceae*. Similarly to other biopolymers, in order to improve chitosan mechanical properties, its performance in terms of water vapor permeability and water solubility the combinations with other biomolecules (pectin, starch, alginate, gelatin) are being investigated providing varied results [32,33].

Polylactic acid (PLA) represents the class of packagings that are synthesized from bio-based monomers. This is one of the most known and commercially available materials. It is fabricated through the polymerization of lactic acid monomer obtained from the fermentation of renewable sources and agricultural wastes (e.g., sugar beets, rice, corn, potato, and wheat starch) [20,27]. The features of PLA include processability, biodegradability, relatively high mechanical strength, thermoplasticity, as well as sufficient moisture barrier [34]. However, large-scale application of this biopolymer is still limited due to its high processing costs, low performance, brittleness, slow degradation rate, and thermal instability [35,36]. PLA is being utilized as a packages and coatings for various food and beverage products such as fruits, vegetables, salads, fresh juices, dairy drinks, yogurts, candies, as well as fishes [36,37,38,39,40,41,42].

One of the most interesting categories of materials applied in packaging industry is represented by the group of biopolymers produced directly by microorganisms. Here, we distinguish e.g., aliphatic polyesters—polyhydroxyalkanoates (PHAs)—and microbial polysaccharides, such as pullulan, curdlan, xanthan and bacterial cellulose. The main advantages of these materials include nontoxicity, biodegradability, plasticity, and selective gas permeability [43,44]. Meanwhile, the major drawbacks of microbial polymers that limit their competitiveness in commercial food packaging applications are both high production costs and relatively poor mechanical properties [44,45]. Currently, polyhydroxyalkanoates are mostly used as a packaging material for food with high oil content (e.g., olives, cheese, nuts), frozen, oxygen-sensitive, as well as organic products [18,46,47,48]. Microbial polysaccharides like pullulan, curdlan, and xanthan offer a good potential for application as edible films and coatings. However, they are most often combined with other polymers and reinforcing additives to improve their physicochemical and mechanical performance [49,50,51,52,53]. Among them, bacterial nanocellulose synthesized as a continuous membrane of desirable shapes, thickness, and size becomes a highly competitive material with a wide range of applications in the packaging industry. All the mentioned biopolymers are presented in Figure 1.

Mostly, the biopackages are recognized as green alternatives to synthetic plastics due to their abundance, nontoxicity, biodegradability, biocompatibility, renewability, and environmental friendliness [54]. They can be used as matrices for incorporating a wide range of reinforcing materials or bioactive compounds, such as antimicrobials, antioxidants, antifungal, nutrients, flavors, colors, etc. [55]. The synthesis of this type of composites is aimed at improving the physicochemical properties of packagings, increasing the quality, and extending the shelf life of foodstuff. However, an industrial application of biopolymers as eco-innovative packaging materials is still limited, not only because of relatively high production costs, but also because of their insufficient mechanical strength, hydrophilic nature, thermal instability, and inadequate barrier properties (e.g., high water vapor and gas permeability). In the latter case, however, various modifications and the development of composite materials give an opportunity to overcome these drawbacks.

In recent years, the growing customers concerns about the environmental pollution and increasing amount of plastics as well as ecological awareness have led to the progress in new green technologies and innovations in the packaging industry. The future-oriented trends of development in this field include active and intelligent systems of packaging. This new generation of materials plays a key role by protecting food products against external influences, enhancing their quality, safety, and shelf-life, or providing information about harmful changes that may occur during the transport and storage. In contrast to traditional packages, active packaging (AP) interacts with packed food and the surrounding environment by means of active agents incorporated into the packaging films. Therefore, the purpose of these materials is to inhibit or delay the mechanisms responsible for foodstuff degradation and spoilage [56]. On the other hand, intelligent packaging (IP, smart packaging) is designed to specifically monitor the conditions of the packaged food and the surrounding environment. This real-time quality system provides information about various factors provoking changes from initial packaging conditions and lowering food quality during its transport and storage [57].

## 3. BNC Production for Commercial Applications

Bacterial nanocellulose is synthesized by acetic acid bacteria in a nutritional culture medium (e.g., Schramm-Hestrin (SH)) via oxidative fermentation. The synthesis may take place in small flasks as well as in larger tanks of any shape. Depending on the bacterial strain and growth time and conditions the wet membranes may achieve the thickness of up to several centimeters. However, the actual process yield, even reaching the highest levels of 6–7 g/L (e.g., for *Ga. hansenii* 53582 it is approximately 2.7–3.0 g/L, for other ATCC collection strains around 1 g/L) is still not satisfactory when industrial-scale operating costs are taken into account [58]. Therefore, the production process remains relatively expensive, which is mainly determined by high costs of cultivation media and other growth factors [12]. The wider commercial applications of BNC, including the food and paper industry, are hampered mainly due to the price [59]. Currently, various studies are being carried out in order to improve bacterial nanocellulose production to reduce operating costs and increase biocellulose synthesis yield [60].

During BNC biosynthesis, the carbon source represents up to 65% of the total costs of this biotechnological process [61]. Therefore, one of the most important and challenging problems limiting the commercial use of bacterial nanocellulose is finding a cost-effective growth medium. At present, many researchers suggest using alternative natural carbon sources (e.g., fruit juices, wheat straw, molasses, maple syrup, cotton-based waste textiles, and other by-products from industry or agriculture) for both economic and ecological reasons [62,63,64,65,66,67,68,69,70]. For instance, Zhao et al. [71] utilized low-cost wine-processing by-products to produce BNC films. Based on the results, it was found that this kind of waste could be a good substrate in fermentation media, which provides sufficient nutrients for the growth of *Ga. xylinus* BC-11 [71]. Rani and Appaiah [72] used coffee cherry husk extract as a carbon source for BNC production by *Ga. hansenii* UAC09. The utilization of this agro-waste has led to a threefold increase in the bacterial nanocellulose biosynthesis yield compared to glucose-rich culture medium [72]. It is strongly believed that the application of appropriate, inexpensive by-products from industry or agriculture will significantly enhance the efficiency of bacterial nanocellulose biosynthesis as compared to the use of a traditional glucose-based medium and at the same time lower the cost of BNC production [72,73,74].

Not only culture medium but also other factors were identified to be important for microbial cellulose biosynthesis enhancement, including the selection of a stably growing BNC-producing strain and rational optimization of culture conditions that ensure high process efficiency [8]. The choice of parameters such as pH, temperature, incubation time, dissolved oxygen content, chemical composition of medium, culture methods (agitated or stationary cultivation), and other operational conditions play the key role in bionanocellulose production [12,60]. It has been shown that supplementation of the defined growth media with alternative substrates (e.g., ethanol, acetic acid, lactic acid, sodium citrate, vitamins, glycerol, polymers such as carboxymethylcellulose (CMC), or agar) can improve BNC biosynthesis [59,75,76,77,78,79,80]. Molina-Ramirez et al. [75] reported the growth in bacterial nanocellulose yield up to 279% by the addition of alternative energy sources, such as ethanol and acetic acid to SH medium. However, the consequence of this supplementation was a deterioration of the structural properties of BNC films (e.g., lowering of crystallinity index and degree of polymerization) [75]. The research of Chen et al. [80] demonstrated a 1.7-fold increase in bacterial nanocellulose yield as a result of 1.5% of CMC application. In addition, the obtained BNC membranes exhibited higher tensile strength and Young’s modulus than the native material [80]. All these activities are intended to increase bionanocellulose production efficiency and thus, to enable successful commercialization of this material e.g., as a food packaging.

One of the most interesting potential approaches to enhance bacterial nanocellulose production as well as to reduce costs of this process is the development of genetic engineered BNC-producing strains [81]. Kuo et al. [82] constructed a mutant of *K. xylinus* by disruption of the gene encoding membrane pyrroloquinoline quinone-dependent glucose dehydrogenase (PQQ GDH) using homology recombination. This GDH knock-out strain could not oxidize glucose to gluconic acid, which resulted in increased glucose conversion to cellulose. The research results showed that the recombinant organism produced BNC with about 40% (stationary culture) and 230% (agitated culture) higher yield when compared to the wild-type strain [82]. Another interesting genetic modification of *K. xylinus* was described by Liu et al. [83]. The heterogeneous expression of *Vitreoscilla* hemoglobin (VHb)-encoding gene in BNC-producing strain allowed to enhance BNC yield by approximately 25% in low-oxygen conditions [83]. Battad-Bernardo et al. [84] showed one of the most impressive examples of the utilization of genetically engineered microorganism, which produced 28-fold more bacterial cellulose from lactose than the parental strain. The recombinant was constructed by the insertion of the promoter-free β-galactosidase (*lacZ*) gene into the wild-type *K. xylinus* ITDI 2.1 strain [84]. However, during BNC synthesis, it should be taken into account that the factors such as bioreactor shape, surface area and medium volume, intensity of shaking, etc., also affect the overall biopolymer synthesis yield and have a significant impact on the properties of the final membranes [61].

## 4. BNC in Food Packaging Applications

Bacterial nanocellulose has a vast range of applications from using as an edible food packaging material to active and/or intelligent food packaging. Generally, biobased packaging should provide chemical, biological, and mechanical protection for the product. In this context, the main advantages of bionanocellulose usage in food industry are edibility, biodegradability, lack of toxicity, good barrier selectivity, and high mechanical strength. BNC as a material generally recognized as safe (GRAS) by the United States Food and Drug Administration (FDA) can be widely used as a safe food ingredient [12]. The main advantage of this material over the other biopolymers is the fact that the stationary bacterial culture offers a full-size, relatively thick membrane, displaying very good mechanical properties and moldability, which only after purification and drying may be ready to use as raw packaging material. However, the application of native microbial cellulose is associated also with drawbacks, such as hydrophilic character of the membranes or lack of antimicrobial and antioxidant activities. To overcome these limitations bacterial nanocellulose is being constantly modified. The most frequent method applies the combination of the natural attributes of the BNC matrix and physicochemical and biological properties of various reinforcing compounds, what allows to improve BNC parameters or to obtain microbial cellulose films with novel, specific characteristics necessary for specific applications. The composite materials consist of a BNC matrix acting as a scaffold and reinforcing compound that imparts its specific physicochemical and biological properties. Generally, the synthesis of BNC composites towards its functionalization and obtaining packaging materials is intended to improve or give new useful features to the native material. The modification of bacterial nanocellulose mainly focuses on the enhancement of physicochemical properties (e.g., degradation abilities, mechanical, thermal, chemical and surface features, rheological characteristics) or introduces a bioactive compounds that preserve, extend, or monitor foodstuff quality and shelf life (active and/or intelligent packagings) [12,85]. A wide range of additives, including antimicrobial and antioxidative agents, nutrients, plasticizers, stabilizers, oxygen scavengers, and antistats may be applied to modify BNC and to develop an active and/or intelligent packaging material [85].

### 4.1. BNC Modifications for the Packaging Industry

**Figure 2:**
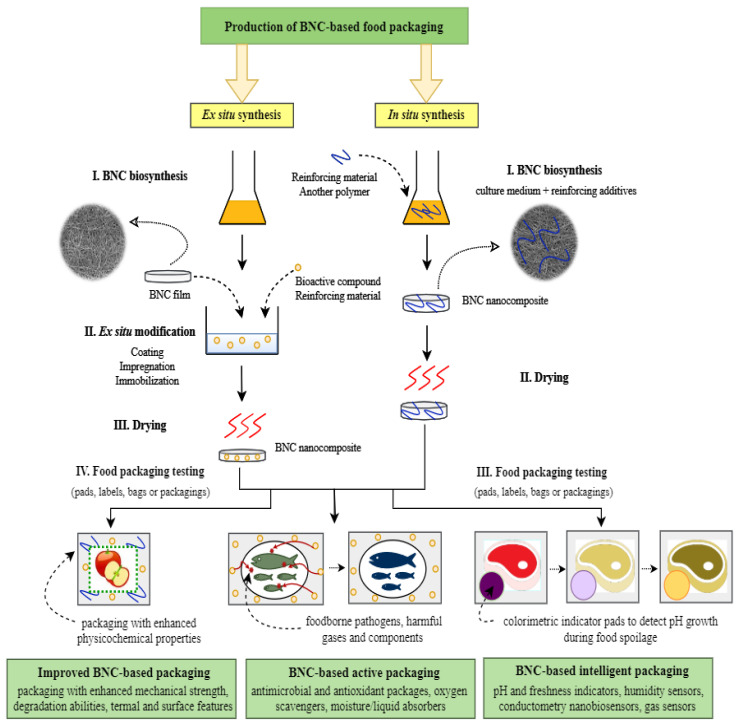
The steps of bacterial nanocellulose (BNC)-based food packaging production.

Composites based on bacterial nanocellulose belong to a relatively new group of materials, which have been formerly proposed for medical applications but can be successfully used in food packaging industry. The introduction of specific functional groups and thus giving the BNC matrix desired properties is possible due to the chemical and physical modifications of this polysaccharide. In the literature, there are two main approaches to bacterial cellulose functionalization: In situ and ex situ methods [86]. The most common strategy used for biopolymer composite synthesis is in situ technique, which involves the addition of reinforcing materials (e.g., sodium alginate, carboxymethylcellulose (CMC), polyvinyl alcohol (PVA), gelatin, agar, pectin, starch) to the culture medium at the beginning of the BNC production process (Figure 2) [80,87,88,89]. The advantages of this modification are the simplicity and the fact that the added compounds become the part of the growing bacterial nanocellulose fibril network, which allows to obtain stable composites with desirable properties. However, the critical limitations of in situ strategy are additives insolubility in culture media, as well as bacterial growth inhibition activity of some reinforcing compounds [86]. In contrast, ex situ modification is carried out after the BNC production process. This post-synthetic strategy is mainly based on the impregnation of a porous, nanofibrillar BNC matrix with bioactive materials (Figure 2). The main advantages offered by the ex situ technique include the possibility of using antimicrobial agents and preserving the original structural features of bacterial nanocellulose. The main drawback of this modification type is the inability to use hydrophobic reinforcing compounds. In addition, only submicron or nano-sized particles can penetrate into bacterial cellulose pores [89]. Nevertheless, these functionalization methods have led to the improvement of the physicochemical properties of native BNC films, which brings new opportunities for their application on the food packaging market.

Native bionanocellulose being itself a food product (known as *Nata de coco*) has been used as a biodegradable edible packaging and can be consumed together with the foodstuff [90,91]. However, owing to its chemical inertness and lack of any reactivity and impact on the surroundings, for most food packaging applications BNC must be modified by the combination with other compounds. These include bioactive agents (antimicrobials, antioxidants, plant extracts, essential oils, organic acids, enzymes etc.), natural pigments, metal ions, inorganic and organic nanoparticles, plasticizers, UV-stabilizers, biopolymers (e.g., chitosan, starch, pectin), or reinforcing additives (e.g., CMC, PVA, polyvinylpyrrolidone (PVP), montmorillonite (MMT)) [8,54]. The result of these actions is the creation of a biomaterial that can protect foodstuff from contamination and external environment (barrier and antibacterial properties) as well as against mechanical damage during the storage and transport (mechanical reinforcement). For instance, the combination of bacterial nanocellulose with PVP and CMC allowed to obtain composites that had better mechanical, optical, and biodegradable properties as compared to native BNC membranes [14]. Therefore, these films could be applied as green alternatives to the conventional food packaging material. BNC/PVP/CMC biocomposites, due to their high tensile strength, provide good resistance to mechanical damage and thus, extend the shelf life of vegetables and meat products [14]. The chemical reactivity of bacterial nanocellulose provided by hydroxyl groups gives a number of possible modifications (in situ during the cultivation process and ex situ) to fulfill specific requirements for the packaging industry (see Figure 2). In addition, high porosity combined with a large surface area make BNC a suitable material for physical interactions with active compounds [12]. Main approaches to the BNC active/intelligent packaging production include impregnation, immobilization techniques, or coating of bacterial nanocellulose matrices [8,90]. For instance, antimicrobial membranes that prevent spoilage caused by foodborne pathogens were obtained by immersing BNC films in e.g., bacteriostatic solutions such as nisin, lactoferrin, or ε-polylysine (see paragraph 4.1.1) [92,93,94]. The methods applying immobilization techniques include, for instance, physical adsorption of an active substance such as fungal laccase and lysozyme or chemical crosslinking by means of e.g., glutaraldehyde (see Table 1) [95,96,97]. Another example of an ex situ modification is the incorporation of silver and alginate-molybdenum trioxide nanoparticles using high-intensity ultrasonic bath into BNC membranes, which were subsequently used as a hybrid film with hydrogen sulfide (H_2_S) sensing ability (see paragraph 4.1.2) [98]. In general, the packaging materials produced according to this technique were designed to maintain food quality.

The BNC-based active and intelligent materials may be used in various forms (e.g., sachets, pads, strips, bags, or as a whole packaging) depending on the intended application (Figure 2). Among others, absorbent pads placed inside packaging are especially used for meat products, because they can remove exuded liquid and thus delay microorganisms’ growth [90]. The BNC-based packing materials can also be divided according to the type of stored product. This material is often used for “dry” food (e.g., pasta, candies, cereal) due to hydrophilic character of this biopolymer, which in contact with liquid absorbs high amounts of water [99]. However, applying specific modifications, the obtained active and intelligent BNC packagings are also designed for meat products, fishes, seafood, mushrooms, fruits, or vegetables [100,101,102,103,104]. The existing literature describes numerous BNC composites with potential use in this sector. A number of them are still at the preliminary testing stage and has not been examined as packaging for specific foodstuff, however the authors suggest their usage in packaging industry [105,106,107].

#### 4.1.1. BNC Active Packagings

**Figure 3:**
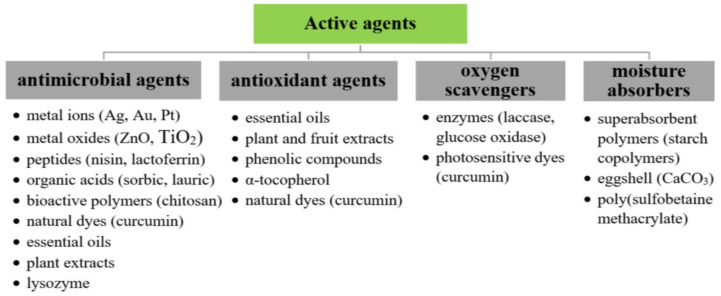
The overview of active compounds applied for BNC active packaging development [54,85,90,111].

Active packaging is a novel group of materials that elongate the shelf life of products through its impact on processes emerging in food such as lipid/protein oxidation, physiological changes, chemical deterioration, microbiological spoilage, and infections caused by insects [104]. This method of packaging films development involves the incorporation of bioactive compounds into the BNC membranes (Figure 3) and was described in several patents [108,109]. In general, different types of active food packagings can be categorized into adsorbing and releasing systems [104]. Emitters are designed to release certain substances (e.g., antimicrobials, antioxidants, carbon dioxide, or flavor and odor emitters) into the packaging content and inhibit adverse processes [90]. Meanwhile, the principal role of absorbers is to remove harmful gases and components from the environment of inside packaging and, therefore, extend the shelf life of the product. These types of materials include liquid and moisture regulators, flavor and odor absorbers, oxygen scavengers, and ethylene absorbers [110].

The development of antimicrobial activity remains a major branch in this field. It includes the combination of a biopolymer with antimicrobial substances. Here, the favorable three-dimensional nanostructure of BNC can act as a matrix and encage biopolymers (e.g., chitosan), antimicrobial agents (e.g., enzymes, nisin), natural preservatives (e.g., plant extracts, essential oils), and inorganic or organic bacteriostatic compounds (e.g., MMT clay, silver, ZnO, TiO_2_) without significantly compromising the primary selective barrier and mechanical properties of packaging films [112]. The effectiveness of the BNC antimicrobial film mainly depends on the choice of bacteriostatic agent, which should be selected according to the type of food packed and deteriorative microbial flora. The literature describes usage of such kind of an active packaging e.g., for storage of meat products in order to minimize the risk of spoilage and poisoning. Bacterial nanocellulose films with adsorbed biomacromolecules such as the already-mentioned nisin or lactoferrin were fabricated to reduce most frequently met foodborne pathogens as *E. coli*, *S. aureus,* and *L. monocytogenes* [92,93,113]. Dos Santos et al. [113] developed composites that could be used in an active food packing system by impregnating BNC films in nisin-EDTA solution. The agar disc diffusion assay showed inhibition zones around the tested samples, which indicated antimicrobial effect against two pathogenic strains *E. coli* and *S. aureus*. Furthermore, it was found that the combination of nisin with chelating agent (EDTA) resulted in even stronger inhibition of bacterial cell wall synthesis. BNC/nisin films also exhibited antioxidant activity, which was examined by DPPH radical scavenging assay [113]. Another approach to produce this type of food packages is the employment of antimicrobial enzymes. Buruaga-Ramiro et al. [97] produced active packaging material through physical immobilization of egg white lysozyme on bacterial nanocellulose matrices. Conducted studies indicated that BNC/Lys films had antimicrobial activity against both Gram-positive (*S. aureus*) and Gram-negative (*E. coli*) bacteria. Immobilization of lysozyme by physical adsorption also caused a statistically significant increase in antioxidant activity of membranes, which could be associated with the enzyme amino groups (NH_2_) that have scavenger ability. In addition, research into the functionality of these biocomposites in the packaging industry showed that they could be stored at room temperature for several weeks without any significant decrease in lysozyme activity [97]. Therefore, BNC/Lys membranes could be potentially applied as a green active packaging material of good stability. Combination of BNC with other polymers was also used to provide antimicrobial properties. Cabañas-Romero et al. [114] produced composites by immersing bacterial nanocellulose in a chitosan solution. The obtained composite material not only exhibited antimicrobial activity against Gram-positive and Gram-negative bacteria, but also showed antioxidant activity and good mechanical properties [114]. Another approach to impart bactericidal features to BNC matrices was the incorporation of metal ions and oxides (e.g., silver, gold, copper, platinum, ZnO, TiO_2_, MgO) [90]. The study of Mocanu et al. [115] presented the synergistic antimicrobial effect of zinc oxide nanoparticles and propolis extracts deposited on BNC. Zinc oxide and other metal oxides nanoparticles exhibited significant activity against food pathogens by generating reactive oxygen species and reducing the integrity of their cell membrane [116]. Organic acids (e.g., lactic, sorbic, lauric etc.) are other examples of antimicrobial compounds widely used in active packaging systems [112]. Zahan et al. [111] designed biodegradable bacterial nanocellulose composites by incorporating lauric acid (LA) as a preservative. The BNC/LA films exhibited antimicrobial activity against *B. subtilis* confirmed during the agar disc diffusion assay [111]. Similar effects of sorbic acid against *E. coli* were observed by Jipa et al. [117]. The antibacterial activity of these organic acids was mainly caused by the decrease in pH, which suppressed microbial growth. The review of BC-based antimicrobial packages and their effect on foodstuff is presented in Table 1.

Apart from microorganisms’ growth, oxidative degradation is another major cause of food spoilage [90]. Therefore, an interesting area of active packaging preparation is the fabrication of antioxidant films. These materials prolong the shelf life of foodstuff by preventing or slowing down the lipid and protein oxidation. Nowadays, the most used antioxidant additives are obtained from food processing wastes and include plant and fruit extracts (e.g., green tea, rosemary, oregano, pomegranate peel), natural compounds (e.g., tocopherol), and essential oils (e.g., citrus, oregano, thyme) [90]. Moradian et al. [100] produced BNC-based active membranes with incorporated pomegranate peel, green tea, and rosemary extract. These natural preservatives, rich in phenolic compounds (i.e., phenolic acids, flavonoids, catechins), in combination with BNC displayed antioxidant properties, therefore the obtained packaging material extended the shelf life of button mushrooms [100]. Curcumin/BNC composite is another example of an active film with antioxidant properties [118]. It consisted of a polyphenolic compound immobilized on bacterial nanocellulose matrix by adsorption method and was found to exhibit good mechanical properties, antibacterial activity, and could be used as pH indicator (see Table 2) [118]. Another example reported by Chen et al. [95] regarded an oxygen scavenger system based on enzyme action. This active packaging material was produced by immobilization of fungal laccase from *Trametes versicolor* on freeze-dried BNC membranes through physical adsorption followed by crosslinking with glutaraldehyde. The research results indicated that the immobilized laccase exhibited higher stability and catalytic activity as compared to the native free enzyme. Furthermore, the enzyme immobilized by crosslinking was active in a broader range of pH and temperature. Therefore, this composite could be used in the packaging industry due to its ability to prevent food deterioration through absorption of oxygen available inside the package [95].

The presence of liquid in a packed food product is another key element that promotes microbial contamination. Different moisture absorbers are often used to control humidity and to remove excess of fluid from foodstuff. Bacterial nanocellulose membranes with eggshell particles were pre-tested in such an application, acting as absorbent material for water and vegetable oil [106]. Vilela et al. [119] produced a BNC/poly(sulfobetaine methacrylate) composite with moisture scavenging ability. This active packaging material extended the shelf life of the product through absorption of fluids released from such foodstuff as fish, meat, fruit, and vegetables [119]. Table 1 provides the summary of BNC-based active packages and their effect on foodstuff.

**Table 1:** The overview of BNC-based active packages and their effect on foodstuff.

Type of BNC Active Packages	Type of Additive	Results	References
**Antimicrobial**	lauric acid (LA)	− inhibition of *B. subtilis* growth	[111]
	poly(sulfobetaine methacrylate)	− antimicrobial activity towards pathogenic microorganisms responsible for food spoilage and foodborne illness (bactericidal activity against *S. aureus* and *E. coli*)	[119]
	silver nanoparticles	− antimicrobial activity against *S. aureus* and *E. coli*	[103]
	ZnO nanoparticles and propolis extracts	− synergistic antimicrobial effect (Gram positive bacteria - *B. subtilis*) and yeast (*C. albicans*)), extended life time of food products	[115]
	ε-polylysine	− antibacterial activity against both *S. aureus* and *E. coli*	[94]
	essential oils	− extension of the shelf life of food through *E. coli* and *L. monocytogenes* growth inhibition	[120]
	bovine lactoferrin	− bactericidal action against food pathogens – *S. aureus* and *E. coli,* used as a bio-based casing for meat products	[93]
	postbiotics of lactic acid bacteria	− antimicrobial activity against *L. monocytogenes,* extension of the shelf life of ground beef at refrigerated storage conditions	[121]
	chitosan	− antimicrobial activity against *S. aureus* and *E. coli*	[118]
	ZnO	− antimicrobial activity against *S. aureus* and *E. coli*	[107]
	TiO_2_	− antimicrobial activity against Gram-negative bacteria	[122]
	sorbic acid	− good antimicrobial activity against *E. coli*	[117]
	lysozyme	− inhibition of *S. aureus, L. monocytogenes, E.coli* and *Y. entrocolitica* growth	[96]
	nisin	− control of *L. monocytogenes* and total aerobic bacteria growth on the surface of vacuum-packaged frankfurters	[92]
**Antioxidant**	green tea extract	− increase of the shelf life, oxidative stability improvement	[105]
	*Echium amoenum* extract	− extension of the shelf life of food products	[123]
	curcumin	− excellent dynamic antioxidant capacity − prevention of lipid oxidation and extension of the shelf life of food products	[118,124]
	propolis extracts	− high antioxidant activity, sustained by the increased content of polyphenols and flavonoids	[115]
	flavonoid silymarin (SMN)	− antioxidant activity, prevention of salmon deterioration and retardation of lipid oxidation − extension of the shelf life of packed fish	[125]
	herbal extracts (rosemary extract)	− preservation and shelf life extension of button mushrooms − oxidative stability improvement	[100]
	*Scrophularia striata* Boiss. extract (SE)	− good antioxidant activity, − controlled release of antioxidant extract	[126]
	lysozyme	− antioxidant activity, extension of the shelf life of packaged food	[97]
**Oxygen scavenger**	laccase	− oxygen scavenging activity, preservation of packed food against deteriorative oxidation processes	[90,95]
**Moisture/liquid absorber**	PVP/CMC	− action as super-absorbent of moisture and fluids (major cause of food spoilage) exuded from packaged fruits − enhancing the shelf life of berries	[101]
	poly(sulfobetaine methacrylate)	− absorption of moisture and water, quality improvement, extension of shelf life through maintaining moisture content	[119]
	eggshell (CaCO_3_)	− water and vegetable oil absorption capacity	[106]

#### 4.1.2. BNC Intelligent Packagings

Intelligent packaging (IP, smart packagings) system represents a new concept that is capable of detecting, identifying, sensing, recording, and/or reporting relevant details about the state and properties of foodstuff. This technology provides information about packaged food, its quality, safety, and changes or irregularities occurring during transport and storage [127]. Compared to active packaging, smart materials do not affect foodstuff to prevent its spoilage, but serve to monitor product conditions. Intelligent packaging includes pH, time-temperature and freshness indicators, humidity detectors, gas and chemical sensors, and numerous other biosensors. One of the most important requirements that must be fulfilled in this kind of smart materials are easy activation and indication of measurable, irreversible changes depending on various factors as well as ideal matching correlated with food quality [110].

**Figure 4:**
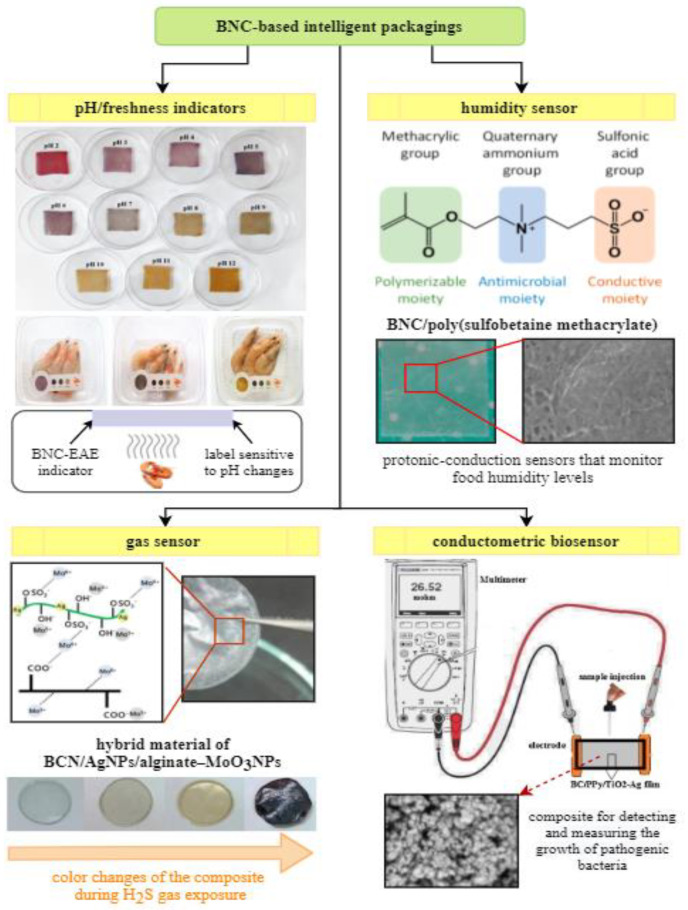
The main types of BNC-based intelligent packagings [98,119,122,123].

Bacterial nanocellulose smart packaging with a pH indicator is one of the recent innovations in food packaging industry. During food spoilage, bacteria produce alkaline metabolites (nitrogen-containing compounds, such as biogenic amines, ammonia, dimethylamine, trimethylamine), which accumulate inside the packaging [127]. Therefore, pH change is a sign of negative changes in foodstuff quality that can be correlated with the growth of pathogenic microorganisms. The detectors of pH changes are mainly composed of BNC support and a dye which is sensitive to pH change. Both natural pigments (e.g., extracted from plants anthocyanins, curcumin) as well as synthetic ones (e.g., bromocresol green/purple, methyl and cresol red, chlorophenol, xylenol, bromothymol blue) are typically used as colorimetric indicators immobilized on biopolymer matrix [127]. The main role of pH detector is to monitor the quality of packaged food and provide qualitative information through visual color changes. Moradi et.al. [128] developed an indicator based on bacterial nanocellulose and black carrot anthocyanins. This intelligent label was fabricated by dip-coating of BNC film into the dye solution. The colorimetric indicator showed an ability to detect pH growth that occur during fish fillets deterioration (freshness sensors). As the pH increased due to food spoilage, films being red at pH 2 became gray in alkaline conditions [128]. Research by Mohammadalinejhad et al. [123] tested colorimetric indicator as a sensor monitoring the freshness of packed shrimps during refrigerated storage. The label was produced by incorporation of natural dye extracted from *Echium amoenum* flowers (EAE) into BNC matrix. The study demonstrated the response of the composite to pH variations in the range between 2 and 12 by color changes from violet to yellow, respectively (Figure 4). The mechanism was based on the fact that the microbial spoilage of protein-rich food generated high levels of volatile nitrogen-based compounds, which lead to pH increase, demonstrated by a color change of the dye immobilized in BNC film [123]. Another example of pH-sensitive indicator was reported by Dirpan et al. [129], where the smart label was created by immersion of BNC films into bromophenol blue solution. The immobilized synthetic pigment responded to pH changes by a color change from dark blue to green and was used as freshness sensor of packed mangoes.

Among others, intelligent packagings comprise gas sensors that are able to detect the presence of gaseous or volatile compounds such as carbon dioxide, oxygen, volatile amines, and other gases indicating the loss of freshness [57]. For instance, Kuswandi et al. [130] developed an intelligent packaging by combining BNC with methyl red that worked as a gas detector based on colorimetric changes resulting from the emission of volatile amines produced during spoilage of foods. In the study by Sukhavattanakul and Manuspiya [98], a hybrid membrane based on BNC loaded with silver (AgNPs) and alginate-molybdenum trioxide nanoparticles (MoO_3_NPs) was fabricated by physical adsorption. The obtained film was used as hydrogen sulfide (H_2_S) sensor. This hazardous gas is generated due to microbial food spoilage and lipids oxidation; therefore, its detection is very useful. The mechanism of action of H_2_S sensor is based on the color reaction that occurs as a result of conversion of Ag to Ag_2_S to create atomic hydrogen, changes in the Mo oxidation state and reduction of MoO_3_NPs to a colored sub-oxide by atomic hydrogen [98].

Maintaining a constant level of humidity inside the food packages is an additional critical point that must be fulfilled to prevent foodstuff deterioration. The increased moisture leads to the growth of pathogenic microorganisms, which affects the quality and shelf life of the product [131]. Vilela et al. [119] fabricated a composite through a free radical polymerization of sulfobetaine methacrylate (SBMA) within the BNC nanofibrous network in the presence of poly(ethylene glycol) diacrylate as a cross-linking agent (Figure 4). This intelligent material acted as humidity sensor to control moisture levels in food products during transportation and storage. The BNC/PSBMA composites possessed protogenic moieties (sulfonic acid groups) in their structure, which were responsible for current conductivity upon exposure to humidity. The system enabled the monitoring of changes in ionic conductivity as a function of liquid content [119].

The application of BNC biosensors in intelligent packaging systems, as mentioned above, has become a very interesting approach for food safety and quality control. This technique, in general, provides the detection of a biological analyte and the subsequent conversion of biochemical signals into an electrical response by means of transducers [57]. To give an example, Ghasemi et al. [122] developed a conductometric nanobiosensor that measured the growth of food pathogenic bacteria (Figure 4). The biosensor was fabricated by the immersion of BNC membrane in TiO_2_–Ag-pyrrole solution, followed by the addition of oxidizing compound (FeCl_3_) to initiate the polymerization of polypyrrole. The main role of this kind of BNC biosensor was to create OH^−^ ions during the interaction between BNC/polypyrrole/TiO_2_–Ag film surface and pathogenic bacteria, what was then recorded as a change in electrical conductivity of the composite [122]. Table 2 presents the overview of BNC-based intelligent materials developed up to date and describes their effect on foodstuff.

**Table 2:** The overview of BNC-based intelligent packagings and their effect on foodstuff.

Kind of BNC Intelligent Packaging	Type of Additive	Results	References
**pH indicator (freshness indicator)**	purple sweet potato anthocyanins	− purple potato anthocyanins change color from red to yellow with the pH change from 2 to 12	[114]
	red cabbage extract (*Brassica oleraceae*)	− color change from bright red to blue (pH range 2–12)	[125]
	*Echium amoenum* extract	− color change from violet to yellow through pH 2–12 − monitoring the freshness of shrimp during refrigerated storage for 4 days − color change with the shrimp age from purple to yellow due to microbial spoilage that leads to pH increase	[123]
	black carrot anthocyanins	− color change from red to gray over the 2–11 pH range − monitoring the freshness (spoilage) of rainbow trout and common carp fillet during the storage at 4°C by means of colorimetric (pH) changes caused by product deterioration	[128]
	curcumin	− color change from yellow to reddish orange depending on the pH caused by the volatile amines evolved from shrimp spoilage in ambient and chiller conditions	[130]
	bromophenol blue	− color change from dark blue (fresh fruit) to green (broken fruit) due to deterioration of mango during ten-day storage	[129]
	methyl red	− detecting pH of broiler chicken pieces by a color change from red to yellow	[102]
**humidity sensor**	poly(sulfobetaine methacrylate)	− protonic-conduction humidity sensing to monitor humidity levels during transport and storage of food product	[119]
**gas sensor**	silver NPs/alginate–molybdenum trioxide nanoparticles (NPs)	− color change from light greyish-white to opaque dark brown black caused by the dissociation of hydrogen from the reaction of AgNPs and H_2_S gas on oxide surfaces	[98]
	methyl red	− detection of volatile amines produced in the package headspace of broiler chicken	[102]
**conductometric nanobiosensor**	polypyrrole/TiO2–Ag	− monitoring of the growth of pathogenic bacteria − measurement of changes in electrical resistance of BC/PPy/TiO2–Ag conducting film used to calculate bacterial growth	[122]
	polypyrrole/ZnO	− monitoring the storage time and temperature of packed chicken thigh by scanning changes in electrical resistance	[132]

## 5. Conclusions and Future Prospects

Packaging is an essential step for food processing that can contribute to quality, safety, shelf-life, convenience, and economic viability of foodstuff. Conventional petroleum-based packaging represents a widely used group of materials mainly due to the low cost, ease of use, and good mechanical properties. However, most of plastics are non-biodegradable and have a number of harmful effects on the environment. Looking from this site, microbial nanocellulose, a bacterially derived, pure biopolymer of glucose, seems to be an excellent ecological alternative to face this problem. Compared with other biopolymers in food packaging sector, BNC can be produced as a full membrane of any shape and size. It also possesses numerous unique properties such as high elasticity, crystallinity and degree of polymerization, chemical purity, and, thanks to ultrafine network structure, outstanding mechanical properties [133]. The environmental friendliness, edibility, biodegradability, nontoxicity, good barrier selectivity, and moldability make bionanocellulose a suitable food packaging. Moreover, the combination of BNC with reinforcing compounds and/or bioactive additives improve or impart new functional physicochemical and biological properties to the biobased material. New technologies of bacterial cellulose active and intelligent food packaging development play an increasingly important role by offering numerous innovative solutions for extending shelf-life and maintaining or monitoring food quality and safety.

In summary, BNC packaging production is currently one of the most dynamically developing trends in the food industry, which has a positive impact on the natural environment, human health, and the quality of stored food product. However, there are still many challenges for commercial low-cost production of BNC-based packaging materials such as low yield of known bacterial nanocellulose strains and relatively high operating costs (e.g., expensive culture media, bioactive agents), especially in comparison with synthetic alternatives. On the other hand, according to the data presented at the Plastic Free World Conference, as much as 62% of European consumers are ready to pay more for packaging containing less plastic. At the same time, many studies have focused on production costs lowering by optimization of culture media composition or replacing the conventional medium (Hestrin and Schramm) by cheaper alternatives including biomass by-products of various industries or agriculture [69]. Similarly, the development of modern molecular and synthetic biology tools provides an opportunity and gives prospects for improving BNC-producing strain through metabolic engineering or heterologous gene expression [81]. Therefore, due to genetic engineering achievements, it is possible not only to intensify bacterial nanocellulose synthesis, but also to extend the range of targeted modifications of this biomaterial for its functionalization. The possible combination of such approaches gives an opportunity to introduce BNC-based packages on the food packaging market as both innovative and functional biopolymer, but also, from a time perspective, practical and price competition for plastic materials.
